# Improved Methods for Thermal Rearrangement of Alicyclic α-Hydroxyimines to α-Aminoketones: Synthesis of Ketamine Analogues as Antisepsis Candidates

**DOI:** 10.3390/molecules17066784

**Published:** 2012-06-04

**Authors:** Hagit Elhawi, Hadar Eini, Amos Douvdevani, Gerardo Byk

**Affiliations:** 1 Laboratory of Nano-Biotechnology, Department of Chemistry, Bar Ilan University, Ramat Gan 52900, Israel; Email: Hagit.Elhawi@sial.com; 2 Nephrology Laboratory, Department of Clinical Biochemistry, Soroka University Medical Center, Ben-Gurion University of the Negev, Beer-Sheva 84101, Israel; Email: einyh@bgu.ac.il (H.E.); amosd@bgu.ac.il (A.D.)

**Keywords:** sepsis, IL-6, TNF-α, thermal rearrangement, α-amino-ketone, microwave

## Abstract

Ketamine is an analgesic/anesthetic drug, which, in combination with other drugs, has been used as anesthetic for over 40 years. Ketamine induces its analgesic activities by blocking the *N*-methyl-D-aspartate (NMDA) receptor in the central nervous system (CNS). We have reported that low doses of ketamine administrated to patients before incision significantly reduced post-operative inflammation as reflected by reduced interleukin-6 (IL-6) sera-levels. Our data demonstrated in a rat model of Gram-negative bacterial-sepsis that if we inject a low dose of ketamine following bacterial inoculation we reduce mortality from approximately 75% to 25%. Similar to what we have observed in operated patients, the levels of TNF-α and IL-6 in ketamine-treated rats were significantly lower than in septic animals not treated with ketamine. On the base of these results, we have designed and synthesized series of new analogues of ketamine applying a thermal rearrangement of alicyclic α-hydroxyimines to α-aminoketones in parallel arrays. One of the analogues (compound **6e**) displayed high activity in down-regulating the levels of IL-6 and TNF-α *in vivo* as compared to ketamine.

## 1. Introduction

Uncontrolled inflammation and immune response lie at the heart of bacterial sepsis. Sepsis is a major complication among post-operative and trauma patients which leads to multi-organ dysfunction, multisystem failure and eventually to death in up to 60% of septic patients in chirurgical intensive care units [1]. The septic response is usually triggered when microorganisms spread from the gastrointestinal tract, skin or lung into contiguous tissues and blood. Animals recognize certain microbial molecules such as lipopolysaccharide (LPS) and rapidly produce various inflammatory mediators including cytokines, such as tumor necrosis factor (TNF) and interleukin-6 (IL-6), that amplify the LPS signal and transmit it to other cells and tissues [2,3]. Inflammatory cytokines amplify and diversify the response. These proteins can exert endocrine, paracrine, and autocrine effects. Blood levels of TNF and IL-6 are high in most patients with severe sepsis. Moreover, intravenous infusion of TNF can elicit many of the characteristic abnormalities of sepsis [4]. 

**Figure 1:**
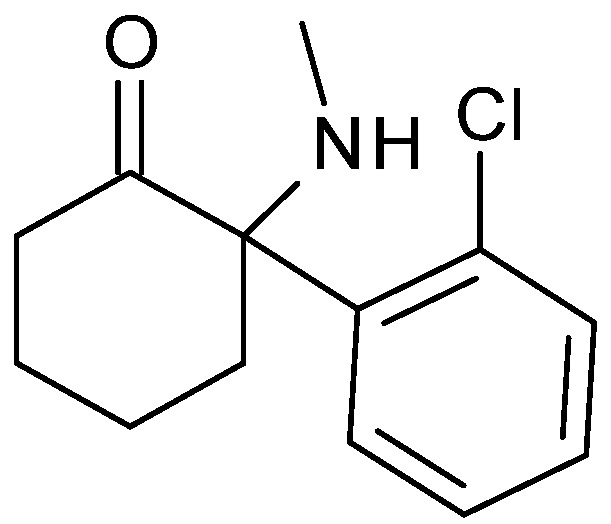
Structure of ketamine.

Current anti-inflammatory pharmacological products such as non-steroidal anti-inflammatory drugs, corticosteroids, antibiotics and anti-cytokines agents have limited efficacy and/or an inadequate safety profiles. Therefore, an intensive effort is currently being made by the scientific community to develop new anti-inflammatory drugs. Ketamine (Figure 1) is an analgesic/anesthetic drug, which, in combination with other drugs, has been used as anesthetic for over 40 years. Ketamine induces its analgesic activities by blocking the *N*-methyl-D-aspartate (NMDA) receptor in the central nervous system (CNS) [5]. 

Studies involving the administration of ketamine to patients and animal have demonstrated an anti-inflammatory activity at a plasma concentration of 0.1–1 µg/mL [6,7]. We have previously shown that ketamine at this concentration had no direct effect on LPS-induced cytokine secretion from leukocytes. Despite these findings, various *in vitro* studies have examined the anti-inflammatory activity of ketamine at concentrations 10- to 1000-fold larger [8,9,10]. We found that at these elevated concentrations ketamine exerts nonspecific cytostatic effects, arrest of cell proliferation, and blockade of cytokine production [11]. Thus, direct inhibitory effect on toll like receptor (TLR) -4 which mediate the effect of LPS is excluded since we found the anti-inflammatory effect of ketamine only “*in-vivo*”. Adenosine mediates the anti-inflammatory effect of ketamine *in vivo*. We previously found that ketamine induced a significant increase in plasma and peritoneal concentrations of the nucleoside adenosine, which occurs within 20–30 min after ketamine administration [12]. Adenosine is an important modulator of inflammation. Its levels rise during ischemia, hypoxia, inflammation and trauma and it exhibits anti-inflammatory effects through A_2A_ receptor (A_2A_R) [13]. We have suggested that the anti-inflammatory effect of ketamine were mediated by adenosine since we found that its effects were blocked by an A_2A_R specific antagonist and were mimicked by an A_2A_ adenosine receptor agonist [10,11,12]. Since ketamine in normal pharmacological doses has no activity *in vitro* we were obligated to use an animal based assay to test the new compounds we have developed. 

Ketamine, a common anesthetic compound used in surgery, is synthesized using a tedious multistep procedure of six consecutives reactions [14,15,16,17], including two thermal rearrangements. Obviously, this classical procedure is not adapted to the synthesis of libraries of ketamine analogues. Herein we propose methods for the fast generation of ketamine analogue libraries in moderate yields.

## 2. Results and Discussion

### 2.1. Chemistry

**Scheme 1:**
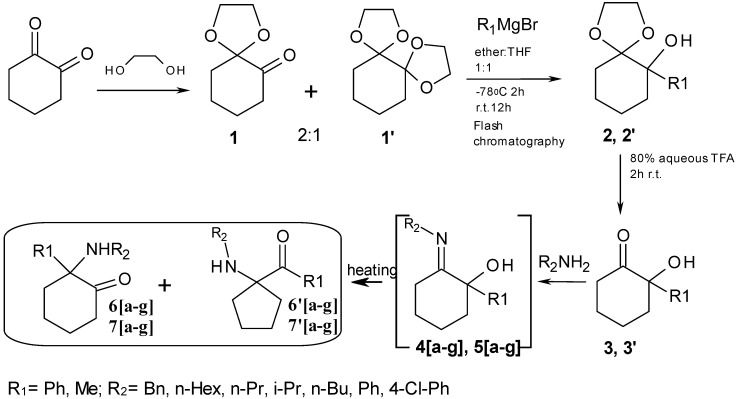
General strategy for the synthesis of ketamines.

Our approach is described in Scheme 1. Intermediate mono-acetal **1a** can be easily obtained after partial conversion of one ketone function in 1, 2-dioxocyclohexane into an acetal using ethylene glycol. Further reaction with appropriate Grignard reagents R^1^MgBr generate alcohols **2** and **2′**, which upon acetal hydrolysis are converted into hydroxyketones **3** and **3′**. The key-steps in this approach are, firstly, the generation of imines (**4a–g**, **5a–g**) and secondly, their rearrangement to mixtures of six-member ring α−aminoketones (**6a–g**, **7a–g**) directly related to ketamine, and five-member ring α−aminoketones (**6′a–g**, **7′a–g**), of potential interest as ketamine isomers bearing the same pharmacophores, but with different structural constrains. A methodology has been developed for obtaining products **6[a–g]**, **7[a–g]**, **6'[a–g]** and **7'[a–g]** in a one pot reaction using both classical or microwave heating of mixtures of the corresponding primary amines R^2^-NH_2_ and the different hydroxyalcohols **3a,b**. In prior works, this type of process needed high temperatures (200 °C) and long reaction times (10–24 h) that were also employed here.

**Figure 2:**
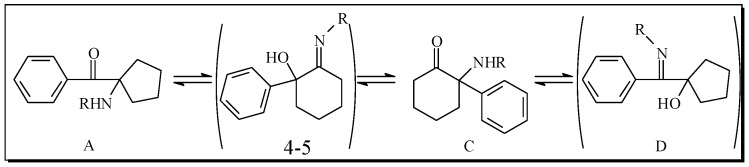
Hypothetical model (according to Stevens [18,19,20,21,22,23,24,25,26]) for inter-conversion between A, C and D by a thermal rearrangement.

This type of thermal rearrangement has been studied for almost half century first by Stevens [18,19,20,21,22,23,24,25,26] and later on by Compain [27,28,29,30], however the conditions needed for the reactions were not improved in those works. Interestingly, our starting compounds **4[a–g]**, **5[a–g]** for the thermal rearrangement were hypothesized (but not isolated) by Stevens (see Figure 2, Figure 4 and Figure 5) as being the intermediates between the starting five member ring A and the product six member ring C isomers in his studies.

**Figure 3:**
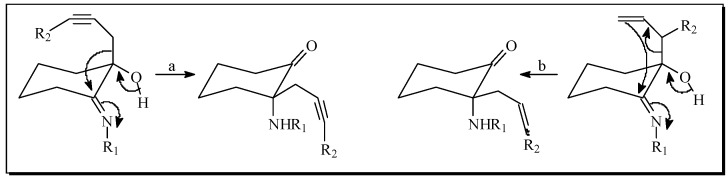
Exo rearrangement in six member ring systems according to Compain [27,28,29,30].

Compain, who also started with six member ring imines (see a in Figure 3), obtained only six membered ring products after rearrangement of the *exo*-propargylic (a in Figure 3) or allylic (b in Figure 3) systems, with no trace of the *endo*-migration that generates five member ring products previously observed by Stevens (see A in Figure 2).

Our results demonstrate experimentally for the first time that the imine intermediates 4-5 (Figure 2) hypothesized (but not observed or isolated) by Stevens as being the intermediate for interconverting A into C and D, are indeed the intermediates between the six and five member rings in the thermal rearrangement. 

Two parallel libraries of analogues were synthesized in a Radley’s parallel reactor using classical heating. The first library with R1 = phenyl **6[a–g]** for the six member ring analogues and **6'[a–g]** for the five member ring analogues, and the second R^1^ = methyl **7[a–g]** for the six member ring analogues and **7'[a–g]** for the five member ring analogues, were generated using a panel of aliphatic and aromatic primary amines R^2^NH_2_ (Table 1).

Overall yields of the product mixtures ranged from low for aromatic amines to good for aliphatic amines. In general, yields were higher for R^1^ = phenyl than for the R^1^ = methyl. The ratio of isomers was favorable to the 6-membered ring adduct for most of the cases where R^1^ = phenyl, however, for R^1^ = methyl the ratio was rather favorable to the 5-membered ring adducts. Isomers were easily isolated using preparative HPLC and fully characterized using a panel of NMR methods and HR-MS and the isolated quantities were large enough for performing substantial biological assays.

**Table 1:** Synthesis of six-member ring compounds **6[a–g]**, **7[a–g]** and five member ring compounds **6'[a–g]**, **7'[a–g]** analogues of ketamine by thermal rearrangement. 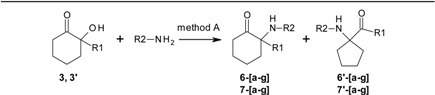

#	R1	R2	Method	Isolated (mg)	Yield %
**6 a**	Ph	Bn	A	104	36
**6'a**	Ph	Bn	A	54	12
**6 b**	Ph	n-hex	A	104	38
**6'b**	Ph	n-hex	A	36	13
**6 c**	Ph	n- pr	A	64	27
**6'c**	Ph	n-pr	A	37	16
**6 d**	Ph	i-pr	A	128	56
**6'd**	Ph	i-pr	A	56	24
**6 e**	Ph	n-Bu	A	142	58
**6'e**	Ph	n-Bu	A	34	14
**6 f**	Ph	Ph	A	20	8
**6'f**	Ph	Ph	A	20	8
**6 g**	Ph	p-Cl-Ph	A	36	12
**6'g**	Ph	p-Cl-Ph	A	96	32
**7 a**	Me	Bn	A	*54	24
**7'a**	Me	Bn	A	*54	24
**7 b**	Me	n-hex	A	60	28
**7'b**	Me	n-hex	A	16	8
**7 c**	Me	n-pr	A	56	32
**7'c**	Me	n-pr	A	68	40
**7 d**	Me	i-pr	A	30	18
**7'd**	Me	i-pr	A	-	0
**7 e**	Me	n-Bu	A	45	24
**7'e**	Me	n-Bu	A	93	51
**7 f**	Me	Ph	A	-	0
**7'f**	Me	Ph	A	20	10
**7 g**	Me	p-Cl-Ph	A	-	0
**7'g**	Me	p-Cl-Ph	A	51	21

* Obtained as a clean mixture.

Further experiments aimed at improving the reaction conditions (time, temperature and yields) were conducted using microwave assisted reactions. A reduced model was chosen using R^2^ = phenyl and a panel of four different R^1^-NH_2_ for which the highest or lowest yields were obtained using the classical thermal conditions (Table 2).

**Table 2:** Synthesis of selected six and five member ring compounds analogues of ketamine by microwave assisted thermal rearrangement.

#	R1	R2	Method	Time reaction (min)	Isolated (mg)	Yield %
**6 a**	Ph	Bn	B	10	84	30
**6'a**	Ph	Bn	B	10	168	60
**6 b**	Ph	n-hex	B	20	54	20
**6'b**	Ph	n-hex	B	20	28	10
**6 c**	Ph	n-pr	B	30	46	20
**6'c**	Ph	n-pr	B	30	46	20
**6 f**	Ph	Ph	B	30	-	0
**6'f**	Ph	Ph	B	30	38	14

To our surprise we could obtain separable mixtures of compounds **6** and **6'** after short reactions. The products were easily separated by preparative HPLC. Interestingly, yields varied for the different amines, while using classical heating method an overall yield of 48% was observed for **6a/6'a** mixtures, the use of microwave improved the yield to 90% with a different distribution of isomers. On the other hand, hexyl- (**6b**, **6'b**) and propyl- (**6c**, **6'c**) amine that gave relatively high yields using classical heating, led to reduced yields with microwave energy. Finally, while reacting aniline (**6f** and **6'f)**, yields were similarly low for both methods, but in contrast to classical heating that resulted in 1:1 isomer mixtures, the microwave reaction led exclusively to isomer **6'f**. Table 3 summarizes the comparative yields obtained by the thermal *vs.* microwave heating methods.

**Table 3:** Yields and six/five ratio of thermal *vs.* microwave assisted rearrangement.

Amine\Parameters	Total yield (Six+Five)		Ratio (Six:Five)	
	Thermal	Microwave	Thermal	Microwave
Benzylamine [**6a**:**6'a**]	48%	90%	3:1	1:2
Hexylamine [**6b**:**6'b**]	51%	30%	3:1	2:1
Propylamine [**6c**:**6'c**]	43%	40%	2:1	1:1
Aniline [**6f**:**6'f**]	16%	14%	1:1	0:1

**Scheme 2:**
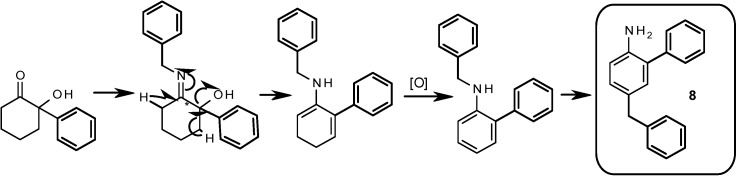
Proposed mechanism for obtaining compound **8**.

A peculiar side compound **8** of synthetic interest has been isolated from the microwave assisted reaction using benzylamine in 8% yield (see Table 3, [**6a-6'a**]). This compound appears to be the result of aromatization of the cyclohexane ring followed by the thermal migration (rearrangement) of the *N*-benzyl group to the *para* position in the generated aromatic system in a similar way to the previously reported photo- [31,32] and microwave- [33] assisted rearrangement of *N*-alkyl anilines (Scheme 2). Compound **8** was unambiguously characterized by NMR and HR-MS. *o-p*-Disubstituted anilines similar to **8** are interesting scaffolds in medicinal chemistry, thus we are currently developing a synthetic methodology for the microwave mediated thermal rearrangement of *o*-substituted *N*-alkyl anilines for generating *o-p*-di substituted anilines.

We conclude that the use of microwaves for the presented thermal rearrangement is advantageous in terms of reaction time (minutes instead of overnight) but not always positive in terms of yield. Strikingly, when using aniline as precursor, classical thermal heating generates a mixture of **6f**/**6'f** (1:1) while the microwave assisted reaction generated exclusively isomer **6'f**. On the base of the works of Stevens and Compain, we speculate that the 5-membered ring is the kinetic product, while the 6-membered ring is the thermodynamic product: when using the classical heating method, equilibrium between both isomers is achieved, however, when performing the reaction for a short time, this equilibrium is not always reached (note that for all the reactions in Table 3 the 6:5 membered ring ratio diminishes favoring the 5-membered ring products when using microwave conditions). Aniline seems to be an extreme case where only the “kinetic” five member ring product is observed. Other mechanistic considerations of this rearrangement will be discussed elsewhere. Finally when using benzylamine as precursor (Table 3, [**6a-6'a**]), a reduced yield of the products is acknowledged accompanied by the presence of side product 8 in 8% yield generated by a tandem elimination-oxidative aromatization of the cyclohexane ring followed by another type of thermal rearrangement of the *N*-benzyl group from the nitrogen to the *p*-position of the generated aromatic ring.

### 2.2. Biological Assays: Inhibitory Effect on IL-6 and TNF-α Secretion

All the compounds were screened for their inhibitory effect on IL-6 and TNF-α secretion in lavage as compared to saline controls and ketamine *in vivo*. We report here results obtained for the active compound **6e** (see Table 4). Treatment with ketamine significantly suppressed the production of IL-6 (p = 0.0450) and TNF-α (p = 0.0158) in lavage after bacterial challenge as compared to saline group.

**Table 4:** Inhibitory effect of compound **6e** on IL-6 and TNF-α secretion in an animal model of sepsis. Compound was injected at 10 mg/kg prior to sepsis (See experimental section for details).

Product	Inhibition ^a-b^ of IL-6	*P value*	Inhibition ^a-b^ of TNF-α	*P value*
Ketamine	35 ± 14	0.0450	30 ± 6.9	0.0158
**6e**	**48 ± 5**	**0.0046**	**37 ± 9**	**0.0069**

^a^ %[lavage /saline]; ^b^ ± %SEM (Ν).

Compound **6e** showed a significant inhibitory effect on IL-6 secretion in lavage as compared to saline group (p = 0.0046). Similarly to the IL-6 results observed in lavage, treatment with compound 6e significantly down-regulated TNF-α levels as compared to saline mice (p = 0.0069). Overall compound 6e with inhibitory effect higher than ketamine for IL-6 and TNF-α secretion appears to be the best candidate for further studies. In addition to cytokines levels, mice behavior was visually monitored following E. coli and compound injection. Sixteen hours after bacterial inoculation mice showed clinical signs of illness such as hunched posture, ruffled fur and reduced activity. Mice treated with ketamine were significantly stronger and more active as compared to mice treated with saline. In concordance to the reduced levels of IL-6 and TNF-α in lavage of **6e** compound-injected mice, these mice showed a similar physical response to the bacterial challenge as the ketamine mice. Overall, we have found that compound **6e** (p = 0.005) has improved inhibitory effect on secretion of IL-6 and TNF-α as compared to ketamine. Moreover, animals treated with this compound were significantly stronger and more active as compared to animals treated with saline.

## 3. Experimental

### 3.1. General Methods

Reagents, unless otherwise mentioned, were purchased from Aldrich and used without further purification. 1,2-Cyclohexanedione was purchased from Acros, Tetrahydrofuran (THF) and diethyl ether (Et_2_O) were distilled from sodium/benzophenone under argon at atmospheric pressure immediately prior to use. Toluene was distilled from calcium hydride under an inert atmosphere and stored over sodium. All other solvents were analytically pure grade and were used without further purification. Analytical and preparative HPLC were performed on a Waters HPLC system equipped with a 717-Plus autosampler, a 600-controller pump, a 996-photodiode array detector and a Gilson 202 fraction collector; the system was operated with the Millennium software (Waters). Selected wavelengths for chromatograms were 220 nm and 254 nm. Mobile phases were (A) H_2_O (0.1% TFA) and (B) MeCN (0.08% TFA). Separation conditions were as follows: Analytical: column C18 Vydac-218TP-54, gradient H_2_O/MeCN, Method A: 3 min [100/0], 3–20 min [50/50], 20–30 min [0/100], 30–40 min [0/100], 41 min [100/0]; flow, 1 mL/min. column Chromolith Performance RP-18e 100–4.6 mm, gradient H_2_O/MeCN, Method B: 1 min [100/0], 1–8 min [0/100], 8–11 min [0/100], 11.1 min [100/0]; flow, 6 mL/min. Preparative: column C18 Vydac-218TP-101510 or column C18 Phenomenex 58 × 21.2 × 10 mm, gradient H_2_O/MeCN. Method D: 3 min [100/0], 3–25 min [50/50], 25–35 min [0/100], 35–50 min [0/100], 51 min [0/100]; flow, 15 mL/min. Method E: 18 min [100/0], 18–40 min [50/50], 40–50 min [0/100], 50–65 min [0/100], 66 min [0/100]; flow, 15 mL/min. Method F: 3 min [100/0], 3–20 min [50/50], 20–30 min [0/100], 30–60 min [0/100], 61 min [0/100]; flow, 15 mL/min.

Preparative normal phase HPLC were performed on a Waters HPLC prep 4000 system equipped with a 4000-controller pump, a 486-absorbance detector with option to one wavelength, manual injector and Gilson 202 fraction collector. Mobile phases were (A) hexane and (B) EtOAc. Separation conditions are as follows: Preparative: column GL Sciences Inc-2GI95001 Inertsil PREP-SIL µm 50 × 250 mm, manual gradient hexane/EtOAc, Method G: 100% hexane until 2:1 hexane/EtOAc (manual change); flow, 70 mL/min.

Microwave irradiations were carried out with a professional “Initiator” microwave from Biotage at pre-fixed temperature. The intensity differed from 0 to 300W at frequency of 2.456 GHz. 

^1^H and ^13^C-NMR spectra were recorded on Bruker DPX-300 and Avance DMX-600 spectrometers. ^1^H-NMR data was obtained by 2D, COSY and HOHAHA methods (t = 40 ms). ^13^C-NMR data was obtained by 2D techniques, NOESY, HMQC and HMBC methods with a delay of 3.45 60 ms respectively in the reverse mode. Chemical shifts are in ppm relative to TMS internal standard or relative to the residual solvent resonance.

Mass spectra analyses were recorded on an AUTOSPEC-FISSONS VG (Micromass) high-resolution mass spectrometer under DCI (desorption chemical ionization) conditions (CH_4_) and by ESI (electron spray ionization) mass spectrometry on a Q-TOF (quadropole time of flight) low-resolution micromass spectrometer (Micromass-Waters, Corp.). Microwave reactions were performed using a mono-mode Initiator station from Biotage. Purity of compounds was over 95% as assessed by two different HPLC conditions, QTOF-MS-MS was used for assessing purity of the molecular peaks. 

### 3.2. Synthesis of Intermediates

*Preparation of 1,4-dioxaspiro[4.5]decan-6-one* (**1**): A solution of 1,2-cyclohexanedione (10 g, 0.089 mol) in toluene (300 mL), an equimolar amount of ethylene glycol (5 mL, 0.089 mol) and *p*-toluenesulfonic acid (100 mg) were heated at reflux for 8 h in a Dean-Stark apparatus (water was continuously separated). The resulting solution was washed twice with 1 N NaOH solution, dried over MgSO_4_ and concentrated under reduced pressure to afford 9.61 g (0.04 mol, 46% yield) of the crude yellow oily product. The crude product containing a certain amount of the diacetal **1′** (2:1 for the monoketone, according to NMR), was used without further purification in the next step. ^1^H-NMR (300 MHz, CDCl_3_) δ 3.93 (m, 4H, H-7', H-7"), 2.47 (m, 2H, H-6), 1.44–1.91 (m, 6H, H-3, H-4, H-5); ^13^C-NMR (75 MHz, CDCl_3_) δ 206.19 (C-1), 106.62 (C-2), 65.05 (C-7', C-7"), 39.51 (C-6), 36.77 (C-3), 26.07 (C-5), 22.55 (C-4); MS(CI+): 157 (MH+); 128 (M-CO). HRMS: *m/z* calc. for C_8_H_12_O_3_ (MH+) 157.0865; found 157.0835.

*Preparation of 6-phenyl-1,4-dioxaspiro[4.5]decan-6-ol* (**2**): To a solution of **1** containing 1 mmol of monoketone in dry THF (25 mL) at −78 °C under N_2_ was added dropwise a solution of phenylMgBr (2 mmol in 50 mL ether) The mixture was stirred at −78 °C and then was warmed to room temperature with stirring overnight. The reaction mixture was quenched by the addition of saturated aqueous NH_4_Cl. The organic layer was removed, and the aqueous layer was extracted twice with ether. The combined organic layers were washed with brine, dried over MgSO_4_, and concentrated under reduced pressure. The residue was purified by preparative normal phase-HPLC (method G, TLC hexane-EtOAc 2:1, vanillin) to give 4.27 g of the desired product as a white solid (60% yield). HPLC analysis Rt = 26.0 min (analytical method A); ^1^H-NMR (600 MHz, CDCl_3_) δ 7.57 (m, 2H, H-2'), 7.29 (m, 2H, H-3'), 7.24 (m, 1H, H-4'), 3.69 (m, 1H, Ha-7''), 3.64 (m, 1H, Ha-7'), 3.34 (m, 1H, Hb-7''), 2.83 (m, 1H, Hb-7'), 2.65 (bs, 1H, OH), 2.30 (m, 1H, Ha-3), 2.12 (m, 1H, Ha-6), 1.79 (m, 1H, Ha-4), 1.78 (m, 1H, Hb-3), 1.74 (m, 1H, Ha-5), 1.64 (m, 1H, Hb-5), 1.59 (m, 1H, Hb-6), 1.55 (m, 1H, Hb-4); ^13^C-NMR (150 MHz, CDCl_3_) δ 143.83 (C-1'), 127.22 (C-3'), 127.05 (C-2'), 126.68 (C-4'), 110.74 (C-1), 76.70 (C-2), 65.52 (C-7"), 65.09 (C-7'), 35.72 (C-3), 32.72 (C-6), 23.26 (C-5), 20.61 (C-4); MS(CI+): 234 (M+); 217 (M-OH+). HRMS: *m/z* calc. for C_14_H_18_O_3_ (M+) 234.1256; found 234.1240.

*Preparation of 2-hydroxy-2-phenylcyclohexanone* (**3**): Compound **2** (0.15 mmol) was treated with aqueous 80% TFA (3 mL) at 0 °C. The reaction mixture was stirred for 3 h at room temperature and then treated with aqueous saturated NaHCO_3_ solution at 0 °C until it became alkaline. The resulting mixture was extracted with CH_2_Cl_2_ (×2). The organic layer was washed with brine, dried over MgSO_4_, and concentrated under reduced pressure to give the desired product as a yellow oil (86–98% yield); HPLC analysis Rt = 22.8 min (analytical method A); ^1^H-NMR (300 MHz, CDCl_3_) δ 7.27 (m, 5H, Ph), 2.91 (m, 1H, Ha-6), 2.40 (m, 2H, Hb-6, Ha-3), 1.57–2.04 (m, 5H, Hb-3, H-4, H-5); ^13^C-NMR (75 MHz, CDCl_3_) δ 211.59 (C-1), 138.92 (C-1'), 128.12 (C-3'), 127.30 (C-4'), 125.37 (C-2'), 79.04 (C-2), 37.88 (C-6), 37.82 (C-3), 27.31 (C-5), 22.04 (C-4); MS(CI+): 190 (M+); 173 (M+-OH); 162 (M+-CO). HRMS: *m/z* calc. for C_12_H_14_O_2_ (M+) 190.0994; found 190.0984.

*Preparation of 6-methyl-1,4-dioxaspiro[4.5]decan-6-ol* (**2′**): Prepared similarly to **2**, but using MeMgBr as Grignard reagent. The residue was purified by silica gel column chromatography (petroleum ether-Et_2_O 3:1 then, petroleum ether-Et_2_O-DCM 2:1:1, vanillin) to give 1.88 g of the desired product as a yellowish oil (60.5% yield). ^1^H-NMR (300 MHz, CDCl_3_) δ 4.00 (m, 4H, H-7', H-7"), 2.19 (bs, 1H, OH), 1.82 (m, 1H, Ha-3), 1.36–1.67 (m, 7H, Hb-3, H-6, H-5, H-4), 1.21 (s, 3H, Me); ^13^C-NMR (75 MHz, CDCl_3_) δ 111.13 (C-1), 73.91 (C-2), 65.60 (C-7"), 65.39 (C-7'), 37.85 (C-6), 31.57 (C-3), 23.32 (C-5), 22.49 (Me), 21.74 (C-4); MS(CI+): 172 (M+); 155 (M+-OH). HRMS: *m/z* calc. for C_9_H_16_O_3_ (M+) 172.1099; found 172.1084.

*Preparation of 2-hydroxy-2-methylcyclohexanone* (**3′**): Prepared similarly to **3** but starting from **2’**. Yellow oil (82–91% yield); ^1^H-NMR (300 MHz, CDCl_3_) δ 3.83 (bs, 1H, OH), 2.54 (m, 2H, H-6), 2.11 (m, 2H, H-3), 1.61–1.85 (m, 4H, H-5, H-4), 1.41 (s, 3H, Me); ^13^C-NMR (75 MHz, CDCl_3_) δ 214.37 (C-1), 76.46 (C-2), 42.05 (C-6), 37.78 (C-3), 27.88 (C-5), 25.07 (Me), 23.01 (C-4); MS (CI+): 129 (MH+); 111 (M-OH). HRMS: *m/z* calc. for C_7_H_12_O_2_ (MH+) 129.0916; found 129.0928.

### 3.3. General Procedure for the Parallel Synthesis of Library Compounds ***6a–g**, **6′a–g***

To seven pressure-tubes each containing 2-hydroxy-2-phenylcyclohexanone (**3**, 190 mg, 1 mmol) was added the appropriate primary amine (benzylamine, hexylamine, propylamine, isopropylamine, butylamine, aniline, 4-chloroaniline, 1.15 mmol). The tubes were flushed with argon, sealed and heated at 200 °C overnight in a Radley’s combinatorial station with stirring. The residues were diluted with ether and extracted with 0.1 M HCl. The acidic phase washed with ether and concentrated and the combined ether phase was dried over MgSO_4_ and concentrated. Preparative HPLC conditions were selected among methods D-F, according to the elution times obtained by analytical HPLC using method A.

### 3.4. Characterization Data for Compounds ***6a–g**, **6′a–g***

For the NMR data numbering system of compounds **6a–g**, **6′a–g** please refer to Table 5.

**Table 5:** Numbering system for NMR chemical shift attribution of library compounds **6a–g**, **6′a–g**.

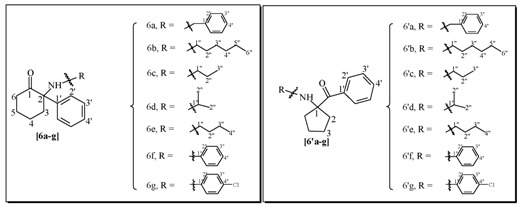

He = equatorial, Ha = axial.

Synthesis of **6a-6′a**: benzylamine was used as amine component in the reaction. The mixture was purified by preparative HPLC using method D to give the desired products. 

*2-(Benzylamino)-2-phenylcyclohexanone* (**6a**): White solid (36% yield); HPLC analysis Rt = 22.3 min (analytical method A); ^1^H-NMR (300 MHz, CDCl_3_ δ 7.54–7.67 (m, 2H, H-2′), 7.54–7.67 (m, 2H, H-3′), 7.54–7.67 (m, 1H, H-4′), 7.46–7.56 (m, 2H, H-3′′), 7.46–7.56 (m, 1H, H-4′′), 7.31–7.42 (m, 2H, H-2′′), 3.78 (d, 1H, CH2-Bn, 12.9), 3.74 (d, 1H, CH2-Bn, 12.9), 3.33 (ddd, 1H, Ha-3, 13.5, 5.7, 2.7), 2.54 (m, 2H, H-6), 2.08–2.22 (m, 1H, Hb-3), 2.08–2.22 (m, 1H, Ha-5), 1.96–2.00 (m, 1H, Ha-4), 1.73–1.88 (m, 1H, Hb-4), 1.73–1.88 (m, 1H, Hb-5); ^13^C-NMR (75 MHz, CD_3_OD) δ 206.96 (C-1), 132.43* (C-1′′), 131.98* (C-4′), 131.51* (C-1′), 131.29* (C-3′), 131.17* (C-3′′), 130.59* (C-4′′), 130.14* (C-2′′), 129.90* (C-2′), 74.28 (C-2), 47.90 (CH2-Bn), 40.47 (C-6), 34.38 (C-3), 28.74 (C-5), 22.80 (C-4); MS(CI+): 280 (MH+); 91 (C7H7+). HRMS: *m/z* calc. for C_19_H_21_NO (MH+) 280.1701; found 280.1734.

*(1-(Benzylamino)cyclopentyl)(phenyl)methanone* (**6′a**): White solid (12% yield); HPLC analysis Rt =23.7 min (analytical method A); ^1^H-NMR (600 MHz, CDCl_3_) δ 8.23 (m, 2H, H-2′), 7.43 (m, 2H, H-3′), 7.52 (m, 1H, H-4′), 7.21 (m, 2H, H-3′′), 7.19 (m, 2H, H-4′′), 7.05 (m, 2H, H-2′′), 3.49 (s, 2H, CH2-Bn), 2.40 (m, 2H, Ha-2), 1.90 (m, 2H, Hb-2), 1.80 (m, 2H, Ha-3), 1.69 (m, 2H, Hb-3); ^13^C-NMR (75 MHz, CDCl_3_) δ 204.30 (COPh), 140.24 (C-1′′), 136.28 (C-1′), 132.17 (C-4′), 129.33 (C-2′), 128.30 (C-3′′), 128.22 (C-2′′), 128.10 (C-3′), 126.99 (C-4′′), 75.17 (C-1), 49.32 (CH2-Bn), 36.68 (C-2), 24.66 (C-3); MS(CI+): 280 (MH+); 174 (M−COPh)+. HRMS: *m/z* calc. for C_19_H_21_NO (MH+) 280.1701; found 280.1726.

Synthesis of **6b-6′b**: Hexylamine was used as amine component in the reaction. The mixture was purified by preparative HPLC using method D to give the desired products.

*2-(Hexylamino)-2-phenylcyclohexanone* (**6b**): Colorless oil (38% yield); HPLC analysis Rt = 25.3 min (analytical method A); ^1^H-NMR (300 MHz, CD_3_OD) δ 7.49–7.61 (m, 2H, H-2′), 7.49–7.61 (m, 2H, H-3′), 7.49–7.61 (m, 1H, H-4′), 3.29 (m, 2H, Ha-3), 2.72 (m, 1H, Ha-1′′), 2.47 (m, 1H, Hb-1′′), 2.47 (m, 2H, H-6), 1.96–2.16 (m, 1H, Hb-3), 1.96–2.16 (m, 1H, Ha-4), 1.96–2.16 (m, 1H, Ha-5), 1.82 (m, 1H, Hb-4), 1.82 (m, 1H, Hb-5), 1.59 (m, 2H, H-2′′), 1.28 (m, 2H, H-3′′), 1.28 (m, 2H, H-4′′), 1.28 (m, 2H, H-5′′), 0.88 (t, 3H, H-6′′, 6.6); ^13^C-NMR (75 MHz, CD_3_OD) δ 207.00 (C-1), 131.77 (C-4′), 131.49 (C-1′), 131.14 (C-3′), 129.59 (C-2′), 73.48 (C-2), 43.47 (C-1′′), 40.24 (C-6), 33.61 (C-3), 32.27 (C-2′′), 28.60 (C-5), 27.24 (C-3′′), 27.20 (C-4′′), 23.32 (C-5′′), 22.74 (C-4), 14.20 (C-6′′); MS(CI+): 274 (MH+); 245 (M-Et)+; 174 (M−NHHex)+. HRMS: *m/z* calc. for C_18_H_27_NO (MH+) 274.2170; found 274.2175.

*(1-(Hexylamino)cyclopentyl)(phenyl)methanone* (**6'b**): Yellowish oil (13% yield); HPLC analysis Rt = 26.3 min (analytical method A); ^1^H-NMR (300 MHz, CD_3_OD) δ 7.86 (m, 2H, H-2′), 7.66 (m, 1H, H-4′), 7.55 (m, 2H, H-3′), 2.96 (m, 2H, H-1′′), 2.61 (m, 2H, Ha-2), 2.15 (m, 2H, Hb-2), 2.15 (m, 4H, H-3), 1.77 (m, 2H, H-2′′), 1.41 (m, 2H, H-3′′), 1.41 (m, 2H, H-4′′), 1.41 (m, 2H, H-5′′), 0.94 (t, 3H, H-6′′, 6.6); ^13^C-NMR (150 MHz, CD_3_OD) δ 199.81 (COPh), 135.06 (C-1′), 134.59 (C-4′), 130.02 (C-2′), 129.87 (C-3′), 79.34 (C-1), 45.63 (C-1′′), 36.31 (C-2), 32.48 (C-2′′), 27.99 (C-3′′), 27.31 (C-4′′), 26.70 (C-3), 23.50 (C-5′′), 14.30 (C-6′′); MS(CI+): 274 (MH+); 168 (M-COPh)+. HRMS: *m/z* calc. for C_18_H_27_NO (MH+) 274.2170; found 274.2184.

Synthesis of [**6c-6′c**]: *n*-Propylamine was used as amine component in the reaction. The mixture was purified by preparative HPLC using method D to give the desired products.

*2-Phenyl-2-(propylamino)cyclohexanone* (**6c**): Yellowish oil (27% yield); HPLC analysis Rt = 21.3 min (analytical method A); ^1^H-NMR (300 MHz, CD_3_OD) δ 7.46–7.62 (m, 2H, H-2′), 7.46–7.62 (m, 2H, H-3′), 7.46–7.62 (m, 1H, H-4′), 3.27 (dd, 1H, Ha-3, 13.8, 5.7, 3), 2.68 (m, 1H, Ha-1′′), 2.34–2.51 (m, 2H, H-6), 2.34–2.51 (m, 1H, Hb-1′′), 1.95–2.19 (m, 1H, Hb-3), 1.95–2.19 (m, 1H, Ha-4), 1.95–2.19 (m, 1H, Ha-5), 1.56–1.82 (m, 1H, Hb-4), 1.56–1.82 (m, 1H, Hb-5), 1.56–1.82 (m, 2H, H-2′′), 0.89 (t, 3H, H-3′′, 7.5); ^13^C-NMR (75 MHz, CD_3_OD) δ 207.03 (C-1), 131.79 (C-4′), 131.44 (C-1′), 131.16 (C-3′), 129.59 (C-2′), 73.44 (C-2), 44.95 (C-1′′), 40.23 (C-6), 33.57 (C-3), 28.61 (C-5), 22.75 (C-4), 20.73 (C-2′′), 11.24 (C-3′′); MS(CI+): 232 (MH+); 203 (M-Et)+; 174 (M-NHPro)+. HRMS: *m/z* calc. for C_15_H_21_NO (MH+) 232.1701; found 232.1697.

*Phenyl(1-(propylamino)cyclopentyl)methanone* (**6'c**): Yellowish oil (16% yield); HPLC analysis Rt = 21.5 min (analytical method A); ^1^H-NMR (300 MHz, CD_3_OD) δ 7.86 (m, 2H, H-2′), 7.66 (m, 1H, H-4′), 7.55 (m, 2H, H-3′), 2.94 (m, 2H, H-1′′), 2.62 (m, 2H, Ha-2), 2.17 (m, 2H, Hb-2), 2.17 (m, 4H, H-3), 1.82 (m, 2H, H-2′′), 1.06 (t, 3H, H-3′′, 7.5); ^13^C-NMR (75 MHz, CD_3_OD) δ 199.82 (COPh), 135.06 (C-1′), 134.59 (C-4′), 130.03 (C-2′), 129.88 (C-3′), 79.31 (C-1), 47.12 (C-1′′), 36.29 (C-2), 26.71 (C-3), 21.47 (C-2′′), 11.34 (C-3′′); MS(CI+): 232 (MH+); 126 (M-COPh)+. HRMS: *m/z* calc. for C_15_H_21_NO (MH+) 232.1701; found 232.1709.

Synthesis of [**6d-6′d**]: *i*-Propylamine was used as amine component in the reaction. The mixture was purified by preparative HPLC using method D to give the desired products.

*2-(Isopropylamino)-2-phenylcyclohexanone* (**6d**): White solid (56% yield); HPLC analysis Rt = 20.0 min (analytical method A); ^1^H-NMR (300 MHz, CD_3_OD) δ 7.50–7.63 (m, 2H, H-2′), 7.50–7.63 (m, 2H, H-3′), 7.50–7.63 (m, 1H, H-4′), 3.39 (sep, 1H, Ha-1′′, 6.6), 2.48 (m, 2H, H-6), 2.23 (ddd, 1H, Ha-3, 12.6, 5.7, 2.7), 2.04–2.16 (m, 1H, Hb-3), 2.04–2.16 (m, 1H, Ha-5), 1.94–1.97 (m, 1H, Ha-4), 1.72–1.86 (m, 1H, Hb-4), 1.72–1.86 (m, 1H, Hb-5), 0.95 (d, 6H, H-2′′, 6.6); ^13^C-NMR (75 MHz, CD_3_OD) δ 207.00 (C-1), 132.00 (C-4′), 131.95 (C-1′), 131.35 (C-3′), 129.87 (C-2′), 74.25 (C-2), 44.58 (C-1′′), 40.23 (C-6), 34.54 (C-3), 28.74 (C-5), 22.78 (C-4), 21.48** (C-2′′), 21.46** (C-2′′); MS(CI+): 232 (MH+); 174 (M−NHiPr)+. HRMS: *m/z* calc.calc. for C_15_H_21_NO (MH+) 232.1701; found 232.1649.

*(1-(Isopropylamino)cyclopentyl)(phenyl)methanone* (**6'd**): Yellowish oil (24% yield); HPLC analysis Rt = 21.0 min (analytical method A); ^1^H-NMR (300 MHz, CD_3_OD) δ 7.85 (m, 2H, H-2′), 7.67 (m, 1H, H-4′), 7.55 (m, 2H, H-3′), 3.44 (sep, 1H, H-1′′, 6.3), 2.64 (m, 2H, Ha-2), 2.27 (m, 2H, Hb-2), 2.06 (m, 4H, H-3), 1.41 (d, 6H, H-2′′, 6.3); ^13^C-NMR (75 MHz, CD_3_OD) δ 200.13 (COPh), 135.25 (C-1′), 134.52 (C-4′), 130.05 (C-2′), 129.87 (C-3′), 80.08 (C-1), 51.87 (C-1′′), 36.63 (C-2), 26.22 (C-3), 21.87 (C-2′′); MS(CI+): 232 (MH+); 126 (M−COPh)+. HRMS: *m/z* calc. for C_15_H_21_NO (MH+) 232.1701; found 232.1646.

Synthesis of [**6e-6′e**]: *n*-Butylamine was used as amine component in the reaction. The mixture was purified by preparative HPLC using method D to give the desired products.

*2-(Butylamino)-2-phenylcyclohexanone* (**6e**): Yellowish solid (58% yield); HPLC analysis Rt = 22.7 min (analytical method A); ^1^H-NMR (600 MHz, CD_3_OD) δ 7.58 (m, 2H, H-3′), 7.58 (m, 1H, H-4′), 7.48 (m, 2H, H-2′), 3.27 (ddd, 1H, Ha-3, 13.8, 6.3, 3), 2.72 (ddd, 1H, Ha-1′′, 12, 10.2, 6), 2.40–2.52 (m, 2H, H-6), 2.40–2.52 (m, 1H, Hb-1′′), 2.04–2.10 (m, 1H, Hb-3), 2.04–2.10 (m, 1H, Ha-5), 1.97 (m, 1H, Ha-4), 1.78 (m, 1H, Hb-4), 1.78 (m, 1H, Hb-5), 1.58 (m, 2H, H-2′′), 1.30 (m, 2H, H-3′′), 0.88 (t, 3H, H-4′′, 7.2); ^13^C-NMR (75 MHz, CD_3_OD) δ 207.08 (C-1), 132.50 (C-1′), 131.85 (C-4′), 131.20 (C-3′), 129.65 (C-2′), 73.52 (C-2), 43.19 (C-1′′), 40.26 (C-6), 33.65 (C-3), 29.32 (C-2′′), 28.67 (C-5), 22.78 (C-4), 20.89 (C-3′′), 13.83 (C-4′′); MS(CI+): 246 (MH+); 217 (M-Et)+; 174 (M−NHBu)+. HRMS: *m/z* calc. for C_16_H_23_NO (MH+) 246.1857; found 246.1828.

*(1-(Butylamino)cyclopentyl)(phenyl)methanone* (**6'e**): Yellowish oil (14% yield); HPLC analysis Rt = 23.9 min (analytical method A); ^1^H-NMR (600 MHz, CDCl_3_) δ 7.87 (m, 2H, H-2′), 7.66 (m, 1H, H-4′), 7.55 (m, 2H, H-3′), 2.97 (m, 2H, H-1′′), 2.65 (m, 2H, Ha-2), 2.24 (m, 2H, Hb-2), 2.11 (m, 4H, H-3), 1.78 (m, 2H, H-2′′), 1.48 (m, 2H, H-3′′), 1.01 (t, 3H, H-4′′, 7.2); ^13^C-NMR (75 MHz, CDCl_3_) δ 199.79 (COPh), 135.05 (C-1′), 134.52 (C-4′), 129.84 (C-2′), 129.78 (C-3′), 79.33 (C-1), 45.40 (C-1′′), 36.28 (C-2), 30.01 (C-2′′), 26.70 (C-3), 20.87 (C-3′′), 13.95 (C-4′′); MS(CI+): 246 (MH+); 140 (M−COPh)+. HRMS: *m/z* calc. for C_16_H_23_NO (MH+) 246.1857; found 246.1818.

Synthesis of [**6f-6′f**]: Aniline was used as amine component in the reaction. The mixture was purified by preparative HPLC using method F to give the desired products.

*2-Phenyl-2-(phenylamino)cyclohexanone* (**6f**): White solid (8% yield); HPLC analysis Rt = 24.3 min (analytical method A); ^1^H-NMR (600 MHz, CDCl_3_) δ 9.46 (bs, 1H##, NH), 7.37 (m, 2H, H-3′), 7.37 (m, 1H, H-4′), 7.18 (m, 2H, H-2′), 7.10 (m, 2H, H-3′′), 7.10 (m, 1H, H-4′′), 6.77 (m, 2H, H-2′′), 2.94 (dq, 1He#, Ha-3, 14, 3.5), 2.51 (dddd, 1He#, Ha-6, 14, 4, 3.5, 2), 2.42 (ddd, 1Ha#, Hb-3, 14, 12.5, 4), 2.35 (ddd, 1Ha#, Hb-6, 14, 12.5, 6) 2.09 (ddqd, 1He#, Ha-5, 13, 6, 4, 3.5), 1.93 (dqdd, 1He#, Ha-4, 13, 4, 3.5, 2), 1.84 (dtdd, 1Ha#, Hb-5, 13, 12.5, 4, 3.5), 1.75 (dtdd, 1Ha#, Hb-4, 13, 12.5, 4, 3.5); ^13^C-NMR (75 MHz, CDCl_3_) δ 206.54 (C-1), 134.50 (C-1′′), 132.20 (C-1′), 130.03 (C-4′), 129.54 (C-3′), 129.01 (C-3′′), 128.46 (C-2′), 126.66 (C-4′′), 123.29 (C-2′′), 73.86 (C-2), 39.22 (C-6), 34.03 (C-3), 27.39 (C-5), 22.01 (C-4); MS(CI+): 266 (MH+); 265 (M+•); 237 (M−Et)+; 93 (NC6H7). HRMS: *m/z* calc.calc. for C_18_H_19_NO (M+) 265.1467; found 265.1494.

*Phenyl(1-(phenylamino)cyclopentyl)methanone* (**6'f**): Yellow-green solid (8% yield); HPLC analysis Rt = 31.6 min (analytical method A); ^1^H-NMR (300 MHz, CDCl_3_) δ 8.07 (m, 2H, H-2′), 7.46 (m, 1H, H-4′), 7.35 (m, 2H, H-3′), 7.11 (m, 2H, H-3′′), 6.80 (m, 1H, H-4′′), 6.80 (bs, 1H##, NH), 6.66 (m, 2H, H-2′′), 2.54 (m, 2H, Ha-2), 2.09 (m, 2H, Hb-2), 1.84 (m, 4H, H-3); ^13^C-NMR (75 MHz, CDCl_3_) δ 203.72 (COPh), 143.07 (C-1′′), 135.57 (C-1′), 132.68 (C-4′), 129.45 (C-2′), 129.15 (C-3′′), 128.33 (C-3′), 120.60 (C-4′′), 116.41 (C-2′′), 74.92 (C-1), 37.47 (C-2), 25.08 (C-3); MS(CI+): 266 (MH+); 265 (M+•); 160 (M−COPh)+. HRMS: *m/z* calc. for C_18_H_19_NO (M+•) 265.1467; found 265.1500.

Synthesis of [**6g-6′g**]: 4-Chloroaniline was used as amine component in the reaction. The mixture was purified by preparative HPLC using method F to give the desired products.

*2-(4-Chlorophenyl)-2-(phenylamino)cyclohexanone* (**6g**): Yellow-brown solid (12% yield); HPLC analysis Rt = 32.2 min (analytical method A); ^1^H-NMR (300 MHz, CDCl_3_) δ 7.58 (bs, 1H##, NH), 7.36 (m, 2H, H-3′), 7.36 (m, 1H, H-4′), 7.25 (m, 2H, H-2′), 7.02 (m, 2H, H-2′′), 6.62 (m, 1H, H-3′′), 3.00 (m, 1H, Ha-3), 2.49 (m, 1H, Ha-6), 2.31 (m, 1H, Hb-3), 2.31 (m, 1H, Hb-6) 1.91 (m, 2H, H-4), 1.91 (m, 2H, H-5); ^13^C-NMR (75 MHz, CDCl_3_) δ 206.81 (C-1), 136.24 (C-1′′), 132.99 (C-1′), 129.46 (C-4′), 129.41* (C-3′), 128.94* (C-3′′), 128.38 (C-4′′), 128.30* (C-2′), 122.24 (C-2′′), 72.30 (C-2), 39.09 (C-6), 35.51 (C-3), 27.73 (C-5), 22.32 (C-4); MS(ES+): 322 (M+Na)+; 300 (MH+); 174 (M−NHC_6_H_4_Cl)+. HRMS: *m/z* calc. for C_18_H_18_NOCl (MH+) 300.1155; found 300.1135.

*(4-Chlorophenyl)(1-(phenylamino)cyclopentyl)methanone* (**6'g**): Yellow-brown solid (32% yield); HPLC analysis Rt = 34.0 min (analytical method A); ^1^H-NMR (300 MHz, CDCl_3_) δ 8.04 (m, 2H, H-2′), 7.44 (m, 1H, H-4′), 7.33 (m, 2H, H-3′), 6.98 (m, 2H, H-3′′), 6.43 (m, 2H, H-2′′), 4.75 (bs, 1H##, NH), 2.55 (m, 2H, Ha-2), 2.00 (m, 2H, Hb-2), 1.81 (m, 4H, H-3); ^13^C-NMR (75 MHz, CDCl_3_) δ 204.43 (COPh), 144.25 (C-1′′), 135.89 (C-1′), 132.34 (C-4′), 128.96* (C-2′), 128.96* (C-3′′), 128.06* (C-3′), 122.51 (C-4′′), 114.89 (C-2′′), 72.82 (C-1), 37.77 (C-2), 24.85 (C-3); MS(CI+): 300 (MH+); 194 (M−COPh)+. HRMS: *m/z* calc.calc. for C_18_H_18_NOCl (MH+) 300.1155; found 300.1164.

### 3.5. General Procedure for the Parallel Synthesis of Library Compounds ***7a–g**, **7′a–g***

To seven pressure-tubes each containing 2-hydroxy-2-methylcyclohexanone (3′, 128 mg, 1 mmol) were added the appropriate primary amine (benzylamine, hexylamine, propylamine, isopropylamine, butylamine, aniline, 4-chloroaniline, 1.15 mmol). The tubes were flushed with argon, sealed and heated at 200 °C overnight in a Radley’s combinatorial station with stirring. The aliphatic residues were concentrated and purified by silica gel column chromatography (100% CH_2_Cl_2_, CH_2_Cl_2_–MeOH 15:0.1 and then CH_2_Cl_2_–MeOH 15:1) to give the desired products. All the cleaned products were treated with 0.1M HCl and washed with ether to give their corresponding hydrochloride salts. 

The aromatic residues were diluted with ether and extracted with 0.1 M HCl. The acidic phase washed with ether several times and the combined ether phases were dried over MgSO_4_ and concentrated. Preparative HPLC were performed with method D, according to the elution times obtained by analytical HPLC using method A.

### 3.6. Characterization Data for Compounds ***7a–g**, **7′a–g***

For the NMR data numbering system of compounds **7a–g**, **7′a–g** please refer to Table 6.

**Table 6:** Numbering system for NMR chemical shifts attribution for library **7a–g**, **7′a–g**.

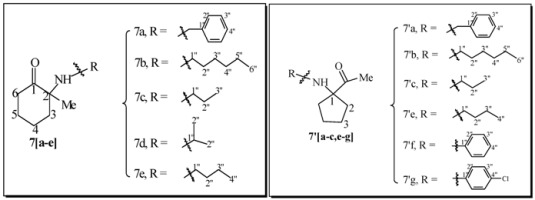

He = equatorial, Ha = axial.

Synthesis of [**7a-7′a**]: Benzylamine was used as amine component in the reaction. The mixture was purified by preparative HPLC using method D to give the desired products as mixtures.

*2-(Benzylamino)-2-methylcyclohexanone + 1-(1-(benzylamino)cyclopentyl)ethanone* (**7a+7′a**). Mixture of products 1:1 as white solid (48% yield); HPLC analysis Rt = 3.4 min (analytical method B); ^1^H-NMR (600 MHz, CD_3_OD) 7a: δ 7.46–7.56 (m, 2H, H-2′), 7.46–7.56 (m, 2H, H-3′), 7.46–7.56 (m, 1H, H-4′), 4.20 (d, 1H, CH2-Bn, 12.6), 4.10 (d, 1H, CH2-Bn, 12.6), 2.82 (td, 1H, Ha-6, 14.4, 6.6), 2.45 (ddt, 1H, Hb-6, 14.4, 4.2, 1.8), 2.28 (ddd, 1H, Ha-3, 12, 6, 3.6), 2.18 (m, 1H, Ha-5), 2.11 (m, 1H, Ha-4), 2.00 (m, 1H, Hb-3), 1.95 (m, 1H, Hb-4), 1.71 (s, 3H, Me), 1.68(m, 1H, Hb-5). **6′a**: δ 7.47–7.56 (m, 2H, H-2′), 7.47–7.56 (m, 2H, H-3′), 7.47–7.56 (m, 1H, H-4′), 4.07 (s, 2H, CH2-Bn), 2.35 (m, 2H, Ha-2), 2.34 (s, 3H, Me), 2.11(m, 2H, Hb-2), 2.02 (m, 4H, H-3); ^13^C-NMR (150 MHz, CD_3_OD) 7a: δ 208.34 (C-1), 132.99* (C-1′), 131.16* (C-3′), 130.71* (C-4′), 130.27* (C-2′), 69.13 (C-2), 47.29 (CH2-Bn), 38.98 (C-6), 36.59 (C-3), 28.85 (C-5), 22.46 (C-4), 19.68 (Me). **7′a**: δ 205.79 (COMe), 131.26* (C-3′), 130.71* (C-4′), 130.32* (C-2′), 130.18* (C-1′), 78.46 (C-1), 49.32 (CH2-Bn), 35.00 (C-2), 27.23 (C-3), 24.45 (Me); MS(CI+): 218 (MH+); 174 (M−COMe)+; 112 (M−NHC7H7+). HRMS: *m/z* calc. for C_14_H_19_NO (MH+) 218.1545; found 218.1561.

Synthesis of [**7b-7′b**]: *n*-Hexylamine was used as amine component in the reaction. The residue was purified by silica gel column chromatography (100% CH_2_Cl_2_, CH_2_Cl_2_–MeOH 15:0.1 and then CH_2_Cl_2_–MeOH 15:1, ninhydrine) to give the desired product. 

*2-(Hexylamino)-2-methylcyclohexanone* (**7b**): Yellow-brown oil (28% yield); ^1^H-NMR (300 MHz, CD_3_OD) δ 2.93 (m, 2H, H-1′), 2.80 (td, 1H, Ha-6, 14.1, 6.3), 2.42 (m, 1H, Hb-6), 2.18 (m, 2H, H-3), 1.94 (m, 1H, Ha-4), 1.94 (m, 1H, Ha-5), 1.90 (m, 1H, Hb-4), 1.90 (m, 1H, Hb-5), 1.68 (m, 2H, H-2’), 1.62 (s, 3H, Me), 1.38 (m, 2H, H-3′), 1.38 (m, 2H, H-4′), 1.38 (m, 2H, H-5′), 0.93 (t, 3H, H-6′); ^13^C-NMR (75 MHz, CD_3_OD) δ 208.53 (C-1), 68.39 (C-2), 43.04 (C-1′), 38.84 (C-6), 36.31 (C-3), 32.44 (C-2′), 27.76 (C-5), 27.76 (C-3′), 27.34 (C-4′), 23.47 (C-5′), 22.14 (C-4), 19.45 (Me), 14.27 (C-6′); MS(CI+): 280 (MH+); MS(CI+): 212 (MH+); 183 (M−Et)+; 154 (M-Bu)+; 112 (M−NHHex)+. HRMS: *m/z* calc. for C_13_H_25_NO (MH+) 212.2014; found 212.1973.

*1-(1-(Hexylamino)cyclopentyl)ethanone* (**7′b**): Yellow oil (8% yield); ^1^H-NMR (300 MHz, CD_3_OD) δ 2.87 (m, 2H, H-1′), 2.29 (s, 3H, Me), 2.28 (m, 2H, Ha-2), 2.02 (m, 2H, Hb-2), 2.02 (m, 4H, H-3), 1.74 (m, 2H, H-2′), 1.37 (m, 2H, H-3′), 1.37 (m, 2H, H-4′), 1.37 (m, 2H, H-5′), 0.93 (t, 3H, H-6′, 6.6); ^13^C-NMR (150 MHz, CD_3_OD) δ 205.80 (COMe), 77.99 (C-1), 45.38 (C-1′), 34.75 (C-2), 32.40 (C-2′), 27.89 (C-3′), 27.25 (C-4′), 27.14 (C-3), 24.36 (Me), 23.45 (C-5′), 14.26 (C-6′); MS(CI+): 212 (MH+); 168 (M−COMe)+. HRMS: *m/z* calc. for C_13_H_25_NO (MH+) 212.2014; found 212.1979.

Synthesis of [**7c-7′c**]: n-propylamine was used as amine component in the reaction. The residue was purified by silica gel column chromatography (100% CH_2_Cl_2_, CH_2_Cl_2_–MeOH 15:0.1 and then CH_2_Cl_2_–MeOH 15:1) to give the desired products. 

*2-Methyl-2-(propylamino)cyclohexanone* (**7c**): Yellow-brown oil (16% yield); ^1^H-NMR (600 MHz, CD_3_OD) δ 2.95 (m, 1H, Ha-1′), 2.85 (m, 1H, Hb-1′), 2.82 (td, 1H, Ha-6, 14.4, 6.6), 2.42 (m, 1H, Hb-6), 2.25 (m, 1H, Ha-3), 2.15 (m, 1H, Ha-5), 1.95 (m, 2H, H-4), 1.92 (m, 1H, Hb-3), 1.73 (m, 2H, H-2′), 1.68 (m, 1H, Hb-5), 1.63 (s, 3H, Me), 1.04 (t, 3H, H-3′, 7.8); ^13^C-NMR (150 MHz, CD_3_OD) δ 208.51 (C-1), 68.37 (C-2), 45.54 (C-1′), 38.86 (C-6), 36.31 (C-3), 27.77 (C-5), 22.27 (C-4), 21.22 (C-2′), 19.48 (Me), 11.34 (C-3′); MS(CI+): 280 (MH+); MS(CI+): 170 (MH+); 112 (M−NHPro)+. HRMS: *m/z* calc. for C_10_H_19_NO (MH+) 170.1545; found 170.1550.

*1-(1-(Propylamino)cyclopentyl)ethanone* (**7'c**): Yellow-brown oil (20% yield); ^1^H-NMR (300 MHz, CD_3_OD) δ 2.85 (m, 2H, H-1′), 2.30 (m, 2H, Ha-2), 2.29 (s, 3H, Me), 2.01 (m, 2H, Hb-2), 2.01 (m, 4H, H-3), 1.76 (m, 2H, H-2′), 1.03 (t, 3H, H-3′, 7.5); ^13^C-NMR (75 MHz, CD_3_OD) δ 205.78 (COMe), 77.93 (C-1), 46.96 (C-1′), 34.74 (C-2), 27.18 (C-3), 24.46 (Me), 21.35 (C-2′), 11.32 (C-3′); MS(CI+): 170 (MH+); 126 (M−COMe)+. HRMS: *m/z* calc. for C_10_H_19_NO (MH+) 170.1545; found 170.1527.

Synthesis of [**7d-7′d**]: iso-propylamine was used as amine component in the reaction. The residue was purified by silica gel column chromatography (100% CH_2_Cl_2_, CH_2_Cl_2_–MeOH 15:0.1 and then CH_2_Cl_2_–MeOH 15:1) to give product [7d]. 

*2-(Isopropylamino)-2-methylcyclohexanone* (**7d**). Yellow oil (18% yield); ^1^H-NMR (300 MHz, CD_3_OD) δ 3.50 (sep, 1H, Ha-1′, 6.6), 2.79 (ddd, 1H, Ha-6, 14.7, 14.1, 6.6), 2.44 (m, 1H, Hb-6), 2.23 (ddd, 1H, Ha-3, 12, 5.7, 3), 1.90–2.18 (m, 1H, Hb-3), 1.90–2.18 (m, 1H, Ha-4), 1.90–2.18 (m, 1H, Hb-4), 1.90–2.18 (m, 1H, Ha-5), 1.90–2.18 (m, 1H, Hb-5), 1.66 (s, 3H, Me), 1.39 (d, 3H, Ha-2′, 6.6), 1.39 (d, 3H, Hb-2′, 6.6); ^13^C-NMR (75 MHz, CD_3_OD) δ 208.25 (C-1), 70.00 (C-2), 49.77 (C-1′), 38.73 (C-6), 36.63 (C-3), 27.49 (C-5), 22.59 (C-4), 22.08 (Me), 22.08 (C-2′), 20.82 (C-2′); MS(CI+): 170 (MH+); 141 (M−Et)+; 126 (M−iPr)+; 112 (M−NHiPr)+. HRMS: *m/z* calc. for C_10_H_19_NO (MH+) 170.1545; found 170.1640.

Synthesis of [**7e-7′e**]: n-butylamine was used as amine component in the reaction. The residue was purified by silica gel column chromatography (100% CH_2_Cl_2_, CH_2_Cl_2_–MeOH 15:0.1 and then CH_2_Cl_2_–MeOH 15:1) to give the desired products. 

*2-(Butylamino)-2-methylcyclohexanone* (**7e**). Yellowish oil (16% yield); ^1^H-NMR (300 MHz, CD_3_OD) δ 2.96 (m, 2H, H-1′, 6.6), 2.80 (td, 1H, Ha-6, 14.4, 6.3), 2.42 (m, 1H, Hb-6), 2.21 (m, 2H, H-3), 1.94 (m, 1H, Ha-4), 1.94 (m, 1H, Ha-5), 1.72 (m, 1H, Hb-4), 1.72 (m, 1H, Hb-5), 1.72 (m, 1H, Ha-2′), 1.63 (s, 3H, Me), 1.48 (m, 1H, Hb-2′), 1.48 (m, 2H, H-3′), 1.00 (t, 3H, H-4′, 7.5); ^13^C-NMR (75 MHz, CD_3_OD) δ 208.53 (C-1), 68.41 (C-2), 42.84 (C-1′), 38.86 (C-6), 36.03 (C-3), 29.77 (C-2′), 27.76 (C-5), 22.15 (C-4), 20.91 (C-3′), 19.50 (Me), 13.95 (C-4′); MS(CI+): 184 (MH+); 140 (M−Pro)+; 126 (M−Bu)+; 112 (M−NHBu)+. HRMS: *m/z* calc. for C_11_H_21_NO (MH+) 184.1701; found 184.1712.

*1-(1-(Butylamino)cyclopentyl)ethanone* (**7'e**). Yellowish oil (34% yield); ^1^H-NMR (300 MHz, CD_3_OD) δ 2.88 (m, 2H, H-1′), 2.30 (s, 3H, Me), 2.28 (m, 2H, Ha-2), 2.01 (m, 2H, Hb-2), 2.01 (m, 4H, H-3), 1.71 (m, 2H, H-2′), 1.44 (m, 2H, H-3′), 0.98 (t, 3H, H-4′, 7.5); ^13^C-NMR (75 MHz, CD_3_OD) δ 205.83 (COMe), 77.97 (C-1), 45.21 (C-1′), 34.76 (C-2), 29.90 (C-2′), 27.17 (C-3), 24.49 (Me), 20.83 (C-3′), 13.93 (C-4′); MS(CI+): 184 (MH+); 140 (M−COMe)+. HRMS: *m/z* calc. for C_10_H_19_NO (MH+) 184.1701; found 184.1738.

Synthesis of [**7f-7′f**]: aniline was used as amine component in the reaction. The organic phase was purified by preparative reverse-HPLC (method D) to give the product [7'f].

*1-(1-(Phenylamino)cyclopentyl)ethanone* (**7'f**). Yellow oil (10% yield); HPLC analysis Rt = 4.9 min (analytical method B); ^1^H-NMR (300 MHz, CDCl_3_) δ 7.20 (m, 2H, H-3′), 6.85 (m, 1H, H-4′), 6.65 (m, 2H, H-2′), 4.36 (bs, 1H, NH), 2.23 (m, 2H, Ha-2), 2.23 (s, 3H, Me), 1.81 (m, 2H, Hb-2), 1.81 (m, 4H, H-3); ^13^C-NMR (75 MHz, CDCl_3_) δ 211.39 (COMe), 142.58 (C-1′), 129.47 (C-3′), 120.68 (C-4′), 116.18 (C-2′), 74.69 (C-1), 35.67 (C-2), 25.27 (Me), 25.08 (C-3); MS(CI+): 204 (MH+); 203 (M+•); 160 (M−COMe)+. HRMS: *m/z* calc. for C_13_H_17_NO (M+•) 203.1310; found 203.1319.

Synthesis of [**7g-7′g**]: 4-Cl-aniline was used as amine component in the reaction. The organic phase was purified by preparative reverse-HPLC (method D) to give the product [7'g].

*1-(1-((3-Chlorophenyl)amino)cyclopentyl)ethanone* (**7'g**). Yellow oil (21% yield); HPLC analysis Rt = 5.8 min (analytical method B); ^1^H-NMR (300 MHz, CDCl_3_) δ 7.48 (bs, 1H, NH), 7.18 (m, 2H, H-2′), 6.70 (m, 2H, H-3′), 2.22 (s, 3H, Me), 2.20 (m, 2H, Ha-2), 1.90 (m, 2H, Hb-2), 1.77 (m, 4H, H-3); ^13^C-NMR (75 MHz, CDCl_3_) δ 219.59 (COMe), 139.78 (C-1′), 129.49 (C-3′), 127.08 (C-4′), 118.50 (C-2′), 75.55 (C-1), 35.34 (C-2), 25.20 (C-3), 24.98 (Me); MS (CI+): 238 (MH+); 194 (M−COMe)+. HRMS: *m/z* calc. for C_13_H_16_NO (MH+) 238.0999; found 238.0957.

### 3.7. Microwave-Assisted Syntheses

Synthesis of [**6a-6′a**]+ compound **8**: To a microwave flask containing 95 mg (0.5 mmol) of 2-hydroxy-2-phenylcyclohexanone 3, was added 0.575 mmol of benzylamine and NMP (1.5 mL). The flask was flushed with argon and sealed. The reaction mixture was heated at 230 °C for 10 min (until starting reagents disappeared according to analytical HPLC method B). The residue was diluted with ten folds of water and TFA was added until clearness. The obtained solution was filtered and purified by preparative reverse-HPLC (method E).

*2-(Benzylamino)-2-phenylcyclohexanone* (**6a**). White solid (30% yield); HPLC analysis Rt = 4.0 min (analytical method B); ^1^H-NMR (300 MHz, CD_3_OD) δ 7.54–7.67 (m, 2H, H-2′), 7.54–7.67 (m, 2H, H-3′), 7.54–7.67 (m, 1H, H-4′), 7.46–7.56 (m, 2H, H-3′′), 7.46–7.56 (m, 1H, H-4′′), 7.31–7.42 (m, 2H, H-2′′), 3.78 (d, 1H, CH2-Bn, 12.9), 3.74 (d, 1H, CH2-Bn, 12.9), 3.33 (ddd, 1H, Ha-3, 13.5, 5.7, 2.7), 2.54 (m, 2H, H-6), 2.08–2.22 (m, 1H, Hb-3), 2.08–2.22 (m, 1H, Ha-5), 1.96–2.00 (m, 1H, Ha-4), 1.73–1.88 (m, 1H, Hb-4), 1.73–1.88 (m, 1H, Hb-5); ^13^C-NMR (75 MHz, CD_3_OD) δ 206.96 (C-1), 132.43* (C-1′′), 131.98* (C-4′), 131.51* (C-1′), 131.29* (C-3′), 131.17* (C-3′′), 130.59* (C-4′′), 130.14* (C-2′′), 129.90* (C-2′), 74.28 (C-2), 47.90 (CH2-Bn), 40.47 (C-6), 34.38 (C-3), 28.74 (C-5), 22.80 (C-4); MS(CI+): 280 (MH+); 91 (C7H7+). HRMS: *m/z* calc. for C_19_H_21_NO (MH+) 280.1701; found 280.1694.

*(1-(Benzylamino)cyclopentyl)(phenyl)methanone* (**6'a**). White solid (60% yield); HPLC analysis Rt = 4.2 min (analytical method B); ^1^H-NMR (300 MHz, CDCl_3_) δ 8.23 (m, 2H, H-2′), 7.43 (m, 2H, H-3′), 7.52 (m, 1H, H-4′), 7.21 (m, 2H, H-3′′), 7.19 (m, 2H, H-4′′), 7.05 (m, 2H, H-2′′), 3.49 (s, 2H, CH2-Bn), 2.40 (m, 2H, Ha-2), 1.90 (m, 2H, Hb-2), 1.80 (m, 2H, Ha-3), 1.69 (m, 2H, Hb-3); ^13^C-NMR (75 MHz, CDCl_3_) δ 204.30 (COPh), 140.24 (C-1′′), 136.28 (C-1′), 132.17 (C-4′), 129.33 (C-2′), 128.30 (C-3′′), 128.22 (C-2′′), 128.10 (C-3′), 126.99 (C-4′′), 75.17 (C-1), 49.32 (CH2-Bn), 36.68 (C-2), 24.66 (C-3); MS(CI+): 280 (MH+); 174 (M-COPh)+. HRMS: *m/z* calc. for C_19_H_21_NO (MH+) 280.1701; found 280.1720.

*5-Benzyl-[1,1'-biphenyl]-2-amine* (**8**). (8% yield). HPLC analysis Rt = 4.8 min (analytical method B); ^1^H-NMR (600 MHz, CDCl_3_) δ 7.41–7.43 (m, 4H, H-2', H-3'), 7.32 (m, 1H, H-4'), 7.27 (m, 2H, H-3''), 7.21 (m, 2H, H-2"), 7.17 (m, 1H, H-4"), 6.98 (m, 1H, H-3), 6.97 (m, 1H, H-5), 6.72 (m, 1H, H-6), 3.91 (s, 2H, CH2-Bn), 3.60 (m, 2H, NH2); ^13^C-NMR (75 MHz, CDCl_3_) δ 144.75 (C-1"), 144.28 (C-1), 139.46 (C-1'), 131.55 (C-4), 130.90 (C-3), 129.09 (C-3'), 128.93 (C-5), 128.81* (C-2"), 128.75* (C-2'), 128.39 (C-3'), 127.91 (C-2), 127.13 (C-4'), 125.88 (C-4"), 116.02 (C-6), 41.02 (CH2-Bn); MS(CI+): 260 (MH+); 259 (M+). HRMS: *m/z* calc. for C_19_H_17_N (M+) 259.1361; found 259.1368.

Synthesis of [**6b-6′b**]: To a microwave flask containing 95 mg (0.5 mmol) 2-hydroxy-2-phenylcyclohexanone 3, was added 0.575 mmol of hexylamine and NMP (1.5 mL). The flask was flushed with argon and sealed. The reaction mixture was heated at 230 °C for 20 min (until starting reagents disappeared according to analytical HPLC method B). The residue was diluted with ten folds of water and TFA was added to clearness. The resulting solution was filtered and purified by preparative reverse-HPLC (method E).

*2-(Hexylamino)-2-phenylcyclohexanone* (**6b**). Yellowish oil (20% yield); HPLC analysis Rt = 4.5 min (analytical method B); ^1^H-NMR (300 MHz, CD_3_OD) δ 7.49–7.61 (m, 2H, H-2′), 7.49–7.61 (m, 2H, H-3′), 7.49–7.61 (m, 1H, H-4′), 3.29 (m, 2H, Ha-3), 2.72 (m, 1H, Ha-1′′), 2.47 (m, 1H, Hb-1′′), 2.47 (m, 2H, H-6), 1.96–2.16 (m, 1H, Hb-3), 1.96–2.16 (m, 1H, Ha-4), 1.96–2.16 (m, 1H, Ha-5), 1.82 (m, 1H, Hb-4), 1.82 (m, 1H, Hb-5), 1.59 (m, 2H, H-2′′), 1.28 (m, 2H, H-3′′), 1.28 (m, 2H, H-4′′), 1.28 (m, 2H, H-5′′), 0.88 (t, 3H, H-6′′, 6.6); ^13^C-NMR (75 MHz, CD_3_OD) δ 207.00 (C-1), 131.77 (C-4′), 131.49 (C-1′), 131.14 (C-3′), 129.59 (C-2′), 73.48 (C-2), 43.47 (C-1′′), 40.24 (C-6), 33.61 (C-3), 32.27 (C-2′′), 28.60 (C-5), 27.24 (C-3′′), 27.20 (C-4′′), 23.32 (C-5′′), 22.74 (C-4), 14.20 (C-6′′); HRMS: *m/z* calc. for C_18_H_27_NO (MH+) 274.2170; found 274.2173.

*(1-(Hexylamino)cyclopentyl)(phenyl)methanone* (**6'b**). Yellowish oil (10% yield); HPLC analysis Rt = 4.7 min (analytical method B); ^1^H-NMR (300 MHz, CD_3_OD) δ 7.86 (m, 2H, H-2′), 7.66 (m, 1H, H-4′), 7.55 (m, 2H, H-3′), 2.96 (m, 2H, H-1′′), 2.61 (m, 2H, Ha-2), 2.15 (m, 2H, Hb-2), 2.15 (m, 4H, H-3), 1.77 (m, 2H, H-2′′), 1.41 (m, 2H, H-3′′), 1.41 (m, 2H, H-4′′), 1.41 (m, 2H, H-5′′), 0.94 (t, 3H, H-6′′, 6.6); ^13^C-NMR (75 MHz, CD_3_OD) δ 199.81 (COPh), 135.06 (C-1′), 134.59 (C-4′), 130.02 (C-2′), 129.87 (C-3′), 79.34 (C-1), 45.63 (C-1′′), 36.31 (C-2), 32.48 (C-2′′), 27.99 (C-3′′), 27.31 (C-4′′), 26.70 (C-3), 23.50 (C-5′′), 14.30 (C-6′′); HRMS: *m/z* calc. for C_18_H_27_NO (MH+) 274.2170; found 274.2190.

Synthesis of [**6c-6′c**]: To a microwave flask containing 95 mg (0.5 mmol) 2-hydroxy-2-phenylcyclohexanone 3, was added 0.575 mmol of propylamine and NMP (1.5 mL). The flask was flushed with argon and sealed. The reaction mixture was heated at 230 °C for 30 min (until starting reagents disappeared according to analytical HPLC method B). The residue was diluted with ten folds of water and TFA was added to clearness. The resulting solution was filtered and purified by preparative reverse-HPLC (method E).

*2-Phenyl-2-(propylamino)cyclohexanone* (**6c**). Yellow oil (20% yield); HPLC analysis Rt = 3.6 min (analytical method B); ^1^H-NMR (300 MHz, CD_3_OD) δ 7.46–7.62 (m, 2H, H-2′), 7.46–7.62 (m, 2H, H-3′), 7.46–7.62 (m, 1H, H-4′), 3.27 (ddd, 1H, Ha-3, 13.8, 5.7, 3), 2.68 (m, 1H, Ha-1′′), 2.34–2.51 (m, 2H, H-6), 2.34–2.51 (m, 1H, Hb-1′′), 1.95–2.19 (m, 1H, Hb-3), 1.95–2.19 (m, 1H, Ha-4), 1.95–2.19 (m, 1H, Ha-5), 1.56–1.82 (m, 1H, Hb-4), 1.56–1.82 (m, 1H, Hb-5), 1.56–1.82 (m, 2H, H-2′′), 0.89 (t, 3H, H-3′′, 7.5); ^13^C-NMR (150 MHz, CD_3_OD) δ 207.03 (C-1), 131.79 (C-4′), 131.44 (C-1′), 131.16 (C-3′), 129.59 (C-2′), 73.44 (C-2), 44.95 (C-1′′), 40.23 (C-6), 33.57 (C-3), 28.61 (C-5), 22.75 (C-4), 20.73 (C-2′′), 11.24 (C-3′′); MS(CI+): 232 (MH+); 203 (M-Et)+; 174 (M−NHPr)+. HRMS: *m/z* calc. for C_15_H_21_NO (MH+) 232.1701; found 232.1714.

*Phenyl(1-(propylamino)cyclopentyl)methanone* (**6'c**). Yellow oil (20% yield); HPLC analysis Rt = 3.7 min (analytical method B); ^1^H-NMR (300 MHz, CD_3_OD) δ 7.86 (m, 2H, H-2’), 7.66 (m, 1H, H-4′), 7.55 (m, 2H, H-3′), 2.94 (m, 2H, H-1′′), 2.62 (m, 2H, Ha-2), 2.17 (m, 2H, Hb-2), 2.17 (m, 4H, H-3), 1.82 (m, 2H, H-2′′), 1.06 (t, 3H, H-3′′, 7.5); ^13^C-NMR (150 MHz, CD_3_OD) δ 199.82 (COPh), 135.06 (C-1′), 134.59 (C-4′), 130.03 (C-2′), 129.88 (C-3′), 79.31 (C-1), 47.12 (C-1′′), 36.29 (C-2), 26.71 (C-3), 21.47 (C-2′′), 11.34 (C-3′′); MS(CI+): 232 (MH+); 126 (M−COPh)+. HRMS: *m/z* calc. for C_15_H_21_NO (MH+) 232.1701; found 232.1673.

Synthesis of [**6f-6′f**]: To a microwave flask containing 95 mg (0.5 mmol) 2-hydroxy-2-phenylcyclohexanone 3, was added 0.575 mmol of aniline and NMP (1.5 mL). The flask was flushed with argon and sealed. The reaction mixture was heated at 230 °C for 30 min (until starting reagents disappeared according to analytical HPLC method B). The residue was diluted with twenty fold of saturated NaHCO_3_ solution and extracted with EtOAc. The organic phase was concentrated, diluted with ether and extracted with 0.1 M HCl. The acidic phase was washed with ether and concentrated. The residue was purified by preparative reverse-HPLC (method E).

Physical properties of *phenyl(1-(phenylamino)cyclopentyl)methanone* (**6'f**). Yellow-brown solid (14% yield); HPLC analysis Rt = 5.4 min (analytical method B); ^1^H-NMR (600 MHz, CDCl_3_) δ 8.07 (m, 2H, H-2′), 7.46 (m, 1H, H-4′), 7.35 (m, 2H, H-3′), 7.11 (m, 2H, H-3′′), 6.80 (m, 1H, H-4′′), 6.80 (bs, 1H##, NH), 6.66 (m, 2H, H-2′′), 2.54 (m, 2H, Ha-2), 2.09 (m, 2H, Hb-2), 1.84 (m, 4H, H-3); ^13^C-NMR (150 MHz, CDCl_3_) δ 203.72 (COPh), 143.07 (C-1′′), 135.57 (C-1′), 132.68 (C-4′), 129.45 (C-2′), 129.15 (C-3′′), 128.33 (C-3′), 120.60 (C-4′′), 116.41 (C-2′′), 74.92 (C-1), 37.47 (C-2), 25.08 (C-3); MS(ES+): 266 (MH+). HRMS: *m/z* calc. for C_18_H_19_NO (M+•) 265.1467; found 265.1483.

### 3.8. Experimental Protocols for Bioassays

To examine the protective effect of the synthesized ketamine derivatives, we used a sepsis model and examined the inflammatory response of treated animals. Sepsis was induced in mice by intra-peritoneal (i.p.) inoculation of a lethal dose of *Escherichia coli* (*E. coli*). Inoculation of animals with live bacteria has been a common tool for studying sepsis mechanisms and represents a model relevant to clinical practice [34,35]. Introduction of bacteria into the peritoneum initiates a rapid response which primarily induces inflammatory and immune cell migration into the infected compartment [35]. Previous data showed a beneficial effect of ketamine on animal survival after *E. coli*-induced sepsis. This effect was attributed to the anti-inflammatory activity of ketamine, as evidenced by decreased IL-6 and TNF-α levels in serum and peritoneal lavage of treated animals [12,13]. To determine the anti-inflammatory effects of ketamine analogues, mice were injected subcutaneously with 10 mg/kg of ketamine or its analogues prior to sepsis induction. As a control, mice were injected with saline. In order to measure systemic and local cytokines levels mice were anesthetized at 16 h after sepsis induction. Blood was drawn by intracardiac puncture and a peritoneal lavage was performed. The concentrations of IL-6 and TNFα were used as inflammation markers and were measured by enzyme-linked immune-sorbent assay (ELISA) as previously described [10]. The ELISA kits that were used are sandwich ELISA kits with 96 well strip plates. These kits are designed for the accurate quantitation of analytes from cell culture supernatant, serum, plasma or other body fluids. In addition, the behavior of mice was observed at time of ketamine analogues injection to confirm that there are no immediate side effects. The analogues-treated mice were also monitored prior to their anesthetization and compared to ketamine and saline treated mice.

#### 3.8.1. Mice, Bacterial Strains, and Drugs

CD1 female mice aged 10 to 12 weeks (Harlan, Jerusalem, Israel) were maintained in the animal laboratory of the Soroka Medical Center. Experiments were done with the permission of the Ben-Gurion University of the Negev Committee for Ethical Care and Use of Animals in Experiments (Beer-Sheva, Israel). E. coli were grown in Luria-Bertani broth (Conda Laboratories, Madrid, Spain) and harvested during the log phase. Bacteria aliquots were stored frozen in LB broth containing 30% glycerol. Ketamine was purchased from Parke Davis (Ketalar, Hampshire, United Kingdom).

#### 3.8.2. Induction of Sepsis and Drug Injection

Ketamine analogues were reconstituted in saline solution to a concentration of 1.25 mg per mL. Sepsis was induced in mice by intra-peritoneal (i.p.) inoculation of a lethal dose of *E. coli* (3.6 × 109 colony forming unit). Prior to bacteria inoculation mice were injected subcutaneously with 200 µL of ketamine (10 mg/kg), its analogues (10 mg/kg) or saline.

#### 3.8.3. Sera and Peritoneal Lavage Fluids Collection and Cytokine Detection

At 16 hours after *E. coli* inoculation, animals were anesthetized with sodium pentobarbital (i.p., 50 mg/kg). A 1 mL syringe flushed with heparin was used to draw intra-cardial blood sample. The samples were stored on ice before centrifugation at 1,000 g at 40 °C for 10 minutes. The cell-free supernatants were collected and frozen at −200 °C for future analysis. Peritoneal lavage was performed with 5 mL phosphate buffer saline (PBS; Biological Industries, Beit Haemek, Israel) containing 2% bovine serum albumin (BSA; Sigma, Rehovot, Israel) and 5 mM ethylenediaminetetraacetic acid (EDTA; Sigma, Rehovot, Israel). After centrifugation at 400 g for 10 minutes, the cell-free supernatants were removed and frozen at −20 °C until analysis. TNFα and IL-6 levels were determined by commercial ELISA kits (Biolegend, San Diego, CA, USA and R&D Systems, Minneapolis, MN, USA, respectively).

### 3.9. Statistical Analysis

Results are expressed as mean ± S.E.M. To compare levels between treated groups and saline group, one-way analysis of variance was used. P values of <0.05 were considered significant.

## 4. Conclusions

We have designed and synthesized libraries of new analogues of ketamine as potential anti-sepsis agents. The synthetic strategy included a thermal rearrangement of alicyclic α-hydroxyimines to five- and six-membered α-aminoketone rings. The use of microwave energy is advantageous in terms of time reactions, but gives ambiguous results in terms of yields. We have confirmed that a α-hydroxy-imine hypothesized by Stevens 40 years ago, is the intermediate of the thermal rearrangement between the five- and six-membered ring ketones. We speculate that the five-membered ring compounds are the kinetic products while the six-membered rings are the thermodynamic products. The compounds were assayed for IL-6 and TNF-α regulation *in vivo*. Analog **6e** was shown to downregulate the levels of IL-6 and TNF-α * in vivo*, which correlates with a normal behavior of mice treated with ketamine in contrast to a hunched posture, ruffled fur and reduced activity in mice treated with inactive compounds or saline. Compound **6e** with a statistically significant improved effect on reduction of IL-6 and TNF-α secretion upon *E. coli* inoculation as compared to ketamine is a good candidate for the treatment of sepsis. Toxicology and other drug-safety *in vivo* studies are on-going.
